# Leukoerythroblastosis as an Unusual Presentation of Parvovirus B19 Infection in a Sickle Cell Patient

**DOI:** 10.1155/2020/8841607

**Published:** 2020-09-22

**Authors:** Pratik A. Patel, Elizabeth P. Weinzierl, Daniel S. Wechsler

**Affiliations:** ^1^Aflac Cancer & Blood Disorders Center, Children's Healthcare of Atlanta, Atlanta, GA, USA; ^2^Department of Pathology, Children's Healthcare of Atlanta, Emory University School of Medicine, Atlanta, GA, USA; ^3^Department of Pediatrics, Emory University School of Medicine, Atlanta, GA, USA

## Abstract

Parvovirus B19 infection in pediatrics most commonly causes fifth disease, a mild viral illness. Hematologic manifestations include severe anemia, especially in patients with chronic hemolytic anemias or who are immunocompromised. Because of the shortened life span of erythrocytes in patients with sickle cell disease, parvovirus infection can cause transient aplastic crisis which can be life-threatening. However, leukocytosis and thrombocytosis are rarely seen. We report leukoerythroblastosis as an unusual presentation of acute parvovirus B19 infection in a previously splenectomized 12-year-old boy with sickle cell disease.

## 1. Introduction

Parvovirus B19 infection is frequently diagnosed in the pediatric population and can be severe in patients with chronic hemolytic anemias such as sickle cell disease. Patients typically present with severe anemia and/or pancytopenia. Here, we describe an unusual presentation of acute parvovirus B19 infection in a patient with sickle cell disease.

## 2. Case Presentation

A 12-year-old boy with sickle cell disease (HbSS) with a history of splenectomy presented to the emergency room for fatigue, headaches, and near syncope. On examination, the patient was tachycardic with a grade 3/6 systolic ejection murmur, marked pallor, and shotty cervical and inguinal lymphadenopathy.

**Figure 1:**
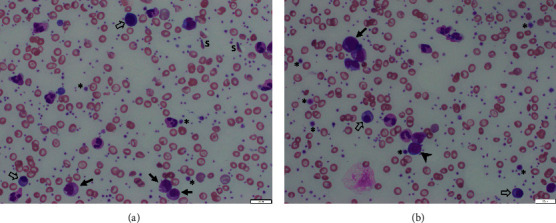
Peripheral blood smear (Wright–Giemsa stain, 50x magnification) showing nucleated red blood cells (open arrows), sickle cells (S), myelocytes (arrows), platelets (asterisks), and a blast (arrowhead). Numerous target cells are also present.

Complete blood count (CBC) showed a white blood cell (WBC) count of 72.2 × 10^9^/L (65.4% neutrophils, 10.3% band forms, 2.7% metamyelocytes, 0.3% myelocytes, 12.3% lymphocytes, 6.7% monocytes, 0.3% eosinophils, and 2% blasts), hemoglobin 3.9 g/dL (baseline hemoglobin 9 g/dL), platelets (PLT) 1533 × 10^9^/L, and 15.3 nucleated red blood cells (RBCs) per 100 WBCs. The reticulocyte count was 1.2%. Morphologic examination of the peripheral blood showed a marked leukoerythroblastosis, rare myeloblasts (∼1%), markedly abundant nucleated RBCs, and thrombocytosis with many large and giant platelets (Figure 1). Lactate dehydrogenase was 772 U/L (normal 157–272 U/L), and uric acid was 5.6 mg/dL (normal 2.6–6.8 mg/dL). Coagulation studies were within normal limits. Of note, ten days earlier, the patient was evaluated at an outside hospital for fever and had a normal CBC : WBC of 12 × 10^9^/L, hemoglobin 9.2 g/dL, and PLT 550 × 10^9^/L.

Based on the presenting CBC, there was concern for a myeloproliferative neoplasm (MPN) such as chronic myeloid leukemia (CML). Peripheral blood flow cytometry showed a myeloid left shift but no abnormal hematolymphoid cell population. Fluorescence in situ hybridization (FISH) testing for the *BCR-ABL1* translocation was negative. CMV and EBV were undetectable by PCR. Because of the severe sudden drop in hemoglobin combined with reticulocytopenia, parvovirus studies were also sent. Parvovirus B19 IgM was positive, and IgG was negative; the parvovirus B19 PCR titer was 6.6 × 10^7^ IU/ml (negative <199 IU/ml) with 1 IU equal to 0.79 copies of parvovirus B19. The patient was transfused with packed red blood cells and received intravenous hydration. Within 48 hours, his WBC count had fallen to 20.7 × 10^9^/L and he was discharged with close outpatient follow-up. Six days later in clinic, his WBC had normalized to 9.35 × 10^9^/L and his PLT count was 1143 × 10^9^/L.

## 3. Discussion

This child with sickle cell disease presented with blood counts concerning for a MPN but was ultimately diagnosed with transient leukoerythroblastosis in the setting of acute parvovirus B19 infection. The diagnosis of acute parvovirus B19 is typically made with serology being positive for IgM and negative for IgG and parvovirus B19 DNA PCR positive [1–3]. While parvovirus B19 viral DNA can be detectable for many months, the titer is usually highest during the acute presentation [4]. Parvovirus B19 infection can span from asymptomatic to life-threatening, depending on the status of the infected host. Patients with sickle cell disease and other chronic hemolytic anemias often develop transient aplastic crisis as a result of acute parvovirus B19 infection [5, 6], which primarily affects erythroid progenitor cells. Other common hematologic manifestations of acute infection in this patient population include reticulocytopenia and leucopenia [1]. Similarly, immunocompromised patients (e.g., those undergoing treatment of leukemia) with parvovirus infection may develop cytopenias as well [7]. Other causes of severe anemia in patients with sickle cell disease include splenic sequestration, acute chest syndrome, hyperhemolysis, and sepsis [8, 9]. Along with severe anemia, parvovirus infection in patients with sickle cell disease classically presents with reticulocytopenia which was seen in this patient. However, the patient also had marked leukocytosis and thrombocytosis, unusual in the setting of parvovirus infection [1, 10, 11]. Leukocytosis >50 × 10^9^/L can be an indication of hematologic malignancy, but is not always associated with a diagnosis of leukemia [12].

Leukoerythroblastosis is a rare condition which is characterized by leukocytosis and the presence of erythroid precursors and myeloid blasts in the peripheral blood. Five cases of parvovirus B19-associated leukoerythroblastosis have been reported [13–17], but all of these patients were neonates and infants (age range preterm neonates to 11 months) and none had thrombocytosis (range 63–250 10^9^/L); in addition, none of them had sickle cell disease. The 12-year-old sickle cell patient described here had an unusual presentation of leukoerythroblastosis in conjunction with transient aplastic crisis secondary to parvovirus B19 infection. The profound thrombocytosis was likely due to a hyperactive marrow, given the degree of anemia on presentation. Patients with splenectomies also tend to have higher platelet counts, including those with chronic hemolytic anemias such as sickle cell disease and hyposplenism [18]. Although Jen and Jackups noted similar findings in a 3-year-old patient with sickle cell disease [19], our patient is the oldest reported patient with leukoerythroblastic reaction in response to parvovirus B19 infection. Disorders such as myelodysplastic syndrome and CML have been temporally associated with concurrent viral infections such as EBV and parvovirus B19 [20–23]. Our patient's presentation suggests that viral serologies and parvovirus B19 DNA PCR should be considered in the workup of older children who present with leukoerythroblastosis.
